# Characterization and Function of Two Short Peptidoglycan Recognition Proteins Involved in the Immunity of *Bactrocera dorsalis* (Hendel)

**DOI:** 10.3390/insects10030079

**Published:** 2019-03-19

**Authors:** Dong Wei, Yu-Wei Liu, Ying-Xin Zhang, Jin-Jun Wang

**Affiliations:** 1 Chongqing Key Laboratory of Entomology and Pest Control Engineering, College of Plant Protection, Southwest University, Chongqing 400716, China; dong_wei1988@yahoo.com (D.W.); yuwei1994@yahoo.com (Y.-W.L.); zyxin1995@163.com (Y.-X.Z.); 2 International Joint Laboratory of China-Belgium on Sustainable Crop Pest Control, State Cultivation Base of Crop Stress Biology for Southern Mountainous Land, Academy of Agricultural Sciences, Southwest University, Chongqing 400715, China

**Keywords:** oriental fruit fly, peptidoglycan, peptidoglycan recognition protein, PGRP, antimicrobial peptide, innate immune

## Abstract

Peptidoglycans (PGNs) are major bacterial components recognized by the immune systems of insects and mammals. PGN recognition proteins (PGRPs) are widely distributed and highly conserved in vertebrates and invertebrates. PGRPs are a family of pattern recognition receptors that recognize peptidoglycan and regulate immune responses. In this study, we cloned two PGRP genes (*BdPGRP-SA* and *BdPGRP-SD*) from *Bactrocera dorsalis* (Hendel), which encode 192 and 196 amino acid residues, respectively. Both genes were highly expressed in adults, especially in the fat body and midgut. These two genes were up-regulated when challenged by the immune triggers, PGN-EB (*Escherichia coli* O111:B4) and PGN-SA (*Staphylococcus aureus*). The suppression of transcriptional expression of either gene by RNA interference (RNAi) resulted in increased sensitivities to Gram-negative *E. coli* and Gram-positive *S. aureus* PGNs. Suppression of *BdPGRP-SA* and -*SD* expression by RNAi resulted in weak expressions of four antimicrobial peptides (AMPs) upon injected with *E. coli* or *S. aureus*. *BdPGRP-SA* and -*SD* are involved in recognizing both Gram-negative and Gram-positive bacteria independently to activate the downstream AMP’s response to bacterial infection.

## 1. Introduction

Insects have an evolutionary conserved innate immune system that protects against pathogen infection [1,2]. The humoral immune reaction is involved in the production of antimicrobial peptides (AMPs), which are mainly synthesized in the fat body and then released into the hemolymph [3]. The expression of AMP genes is regulated by the Toll and immune deficiency (Imd) signal transduction pathways. The Toll pathway is activated by Gram-positive bacteria and fungi, while the Imd pathway is triggered by Gram-negative bacteria [4,5]. Microbial recognition represents the first step of the immune response. For pathogen detection, innate immunity functions with receptors that recognize the conserved motifs of pathogens (peptidoglycan = PGN) but that are absent in the host [6]. PGN is recognized by conserved host PGN recognition proteins (PGRPs). All Gram-negative and a subset of Gram-positive bacteria (e.g. *Bacillus*) possess a meso-diaminopimelic acid (DAP) residue at the third position of the peptide bridge, whereas Gram-positive bacteria possess a lysine (Lys) [7,8,9,10].

*Drosophila* can discriminate between bacteria containing either Lys-type PGN (Lys-PGN) and DAP-type PGN (DAP-PGN) to elicit distinct antimicrobial responses via the selective activation of the Toll and Imd pathways, respectively [7]. Lys-PGN Gram-positive bacteria can be recognized by the secreted PGRP-SA, which activates the Toll pathway to stimulate the expression of antimicrobial peptide genes (e.g., Drosomycin) via the NF-κB members Dif and Dorsal [11,12]. In contrast, DAP-PGN Gram-positive bacteria trigger the Imd pathway through the NF-κB transcription factor Relish. For example, PGRP-SD acts upstream and activates the Imd pathway in *Drosophila* [13]. In insects, PGRPs are classified into short (PGRP-S) and long (PGRP-L) types according to their size. In *D. melanogaster,* there are six short PGRPs: *PGRP-SA*, *-SB*, *-SC1A*, *-SC1B*, *-SC2*, and *-SD* [14]. The short PGRPS are extracellular proteins prefixed with signal peptides, widely present in the hemolymph, cuticle, and fat body cells, and sometimes in epidermal cells of gut and hemocytes [10,15].

In *Drosophila*, some PGRPs function as enzymatic PGRPs (e.g., PGRP-SB and -SC), by removing the peptide from the glycan chains and cleaving it into non-immunized fragments [16,17]. Non-catalytic PGRP lost enzymatic activity but retained the ability to bind peptidoglycans, mediating microbial ligand-dependent downstream signaling [18]. For example, PGRP-SA activates the Toll pathway in response to Gram-positive bacterial infection in *Drosophila* [19,20]. The PGRPs independent functions in two pathways were also observed in *B. mori*. For example PGRP-L6, has a crucial role in Imd pathway activation [21], while PGRP-S5 negatively regulates AMP’s production in an amidase-dependent fashion via the Imd pathway [22]. PGRP-SD can bind Gram-positive bacteria to activate the Toll pathway [20,23], and also act as a recognition protein that is required upstream of the Imd pathway for defense against Gram-negative bacteria [24,25]. The functions of PGRP-SA and -SD are complex and important in insect response to bacterial infection.

The oriental fruit fly, *Bactrocera dorsalis* (Hendel), attacks a variety of commercial fruits and vegetables [26]. To date, no studies have documented the PGRPs and their functions in the *B. dorsalis* immune system. Here, we cloned two short PGRPs (*PGRP-SA* and *PGRP-SD*) in *B. dorsalis*. Their expressions in different developmental stages and adult tissues were analyzed using RT-qPCR. Their functions were further investigated using RNA interference (RNAi). This study provided some basic information on immunity in *B. dorsalis.*

## 2. Materials and Methods

### 2.1. Insects

The laboratory strain of *B. dorsalis* was originally obtained from Haikou, Hainan province, China, in 2008, and was reared at 27.5 ± 0.5 °C with a relative humidity of 70 ± 5% under a 14:10 h (L:D) photoperiod. Newly laid eggs were transferred to an artificial diet as described previously [27,28].

### 2.2. Total RNA Extraction and First Strand cDNA Synthesis

The total RNA was extracted using TRIzol reagent (Invitrogen, Carlsbad, CA, USA) from *B. dorsalis* female adults following manufacturer instructions. RNA was quantified with a NanoDrop One (Thermo Fisher Scientific, Madison, WI, USA), and the quality was evaluated via the absorbance ratio of optical density OD_260/280_ and OD_260/230_. To eliminate genomic DNA, the total RNA samples were treated with RQ1 DNaseI (Promega, Madison, WI, USA). Then first-strand cDNA was synthesized using PrimeScript^®^ RT reagent Kit (Takara, Dalian, China) according to the standard manufacturer protocol. Briefly, the reaction volume was 10 µL, which contained ≈500 ng of total RNA, 200 pmol of random hexamers, 2 µL of reverse transcription buffer, 0.5 µL of reverse transcriptional enzyme mix, and additional RNase-free H_2_O to a final volume of 10 µL. The reverse transcription reaction was performed on a C1000TM Thermal Cycler (Bio-Rad, Hercules, CA, USA) at 37 °C for 15 min followed by 85 °C for 5 s.

### 2.3. Cloning of BdPGRP-SA and BdPGRP-SD

The fragments of two genes (Bd*PGRP*-*SA* and Bd*PGRP*-*SD*) were obtained from the transcriptomes of *B. dorsalis* [29]. The deduced amino acid sequences of selected PGRPs genes were predicted using the online open reading frame (ORF) Finder (http://www.ncbi.nlm.nih.gov/gorf/orfig.cgi). Two pairs of gene-specific primers were designed using DNAMAN 7.0 (Lynnon Biosoft, QC, Canada) to amplify the ORFs (Table S1). PCR was carried out in a 25 µL reaction volume using high fidelity DNA polymerase PrimerSTAR kit (TaKaRa) with the following procedure: 98 °C for 2 min, and then 35 cycles of 95 °C for 30 s, 60 °C for 30 s, and 72 °C for 1 min, followed by a final step of 72 °C for 10 min. The PCR products were checked on a 1.0% gel after electrophoresis and then purified using a gel extraction kit (Takara). The purified fragments were cloned into pGEMT Easy Vector (Promega) and transformed into DH5α competent cells (Biomed, Beijing, China). We used Luria-Bertani agar plates containing 0.1% ampicillin to select the positive transformants for sequencing (BGI, Beijing, China).

### 2.4. Characterization, Sequence Alignment, and Phylogenetic Analysis

The amino acid sequences of both *PGRPs* were predicted using Primer Premier 5.0 software (Premier Biosoft, Palo Alto, CA, USA). The potential PGRP protein sequences were further analyzed for the presence of putative functional domains using InterProScan 5 (https://www.ebi.ac.uk/interpro), for signal peptides prediction using SignalP 4.1 Server (http://www.cbs.dtu.dk/services/SignalP), and for transmembrane domains prediction using TMHMM Server 2.0 (http://www.cbs.dtu.dk/services/TMHMM-2.0). The homologous sequences from other insect species, including *D. melanogaster*, *Tribolium castaneum*, *Culex quinquefasciatus*, *Anopheles gambiae*, and *B. mori* were obtained from the NCBI database via BLAST. The sequence alignment of Bd*PGRP-SA* and Bd*PGRP-SD* with the homologs from other insects were performed using Clustal omega and visualized using Jalview 2.0 software [30]. The phylogenetic tree of amino acid sequences of the PGRPs was constructed using a neighbor-joining method with MEGA 5.0 with 1000 bootstrap replicates [31].

### 2.5. Quantitative Real-Time Polymerase Chain Reaction (qRT-PCR)

To determine the expression profiles of *BdPGRP-SA* and *-SD* in different developmental stages and tissues, we sampled eggs; 1-, 3-, 5-, and 8-day-old larvae; 1-, 3-, 5-, and 7-day-old pupae; and 1-, 3-, 5-, and 7-day-old adults, as well as various body parts or tissues from 5-day-old adults. Several individuals were pooled together into one replicate. The adult central nervous system, midgut, fat body, Malpighian tubules, hindgut, ovary, and testis were dissected and used for total RNA isolation as above. After preparation of templates, a CFX384 Optics Module (Bio-Rad, Singapore) was used for qRT-PCR. Each 10 µL of PCR reaction mixture consisted of 5 µL of Novostar-SYBR Supermix (Novoprotein, Shanghai, China), 3.5 µL of nuclease-free water, 0.5 µL of cDNA (≈500 ng/µL), and 0.5 µL each of forward and reverse primers (10 µM). The qPCR condition was as follows: initial denaturation at 95 °C for 2 min, followed by 40 cycles of 95 °C for 15 s and 60 °C for 30 s. At the end of the procedure, a melting curve analysis from 60 to 95 °C was recorded to ensure the specificity and consistency of all generated products. *Alpha-tubulin* (GenBank: GU269902) was used as an internal reference gene based on previous evaluations [32]. A standard curve was established for each set of primers to validate their specificity. All of the experiments were performed in three biological replicates, and the data were analyzed using the comparative 2^−ΔΔCT^ method [33].

### 2.6. Immune Triggers Induction

The peptidoglycan of PGN-EB from the Gram-negative *E. coli* O111:B4 (Invivogen, San Diego, CA, USA) and PGN-SA from the Gram-positive *Staphylococcus aureus* (Invivogen) were used to test the response of *BdPGRP-SA* and *BdPGRP-SD* to microbial pathogen infection in *B. dorsalis*. PGN-EB, purified from *E. coli*, and PGN-SA, purified from the *S. aureus*, were diluted in 1× PBS (pH 7.2) to a final concentration of 100 ng/μL. For stimulus and transcriptional analysis, 5-d-old adult *B. dorsalis* were injected with 200 nL of PGN-SA and PGN-EB solutions, respectively. Flies injected with 200 nL PBS only were the negative control. Treated flies were collected at 3, 6, 9, 12, and 24 h after injection. All of the samples were collected for total RNA extraction immediately as described above for gene expression determination using qRT-PCR. Three replicates at each time point were performed.

### 2.7. RNA Interference (RNAi)

RNAi was used to explore the potential function of *BdPGRP-SA* and *BdPGRP-SD* in *B. dorsalis*. The unique nucleotide regions of *BdPGRP-SA* and -*SD* were selected for dsRNA synthesis using gene-specific primers (Table S1). DsRNAs of *BdPGRP-SA* and -*SD* were synthesized using a TranscriptAid T7 High Yield Transcription Kit (Thermo Scientific, Vilnius, Lithuania). Agarose gel electrophoresis was used to measure the size and quality of dsRNA and the NanoDrop One spectrophotometer was used to test the dsRNA concentration. The dsRNA of *GFP* was used as the negative control [34]. Approximately 1.2 µg of ds*PGRPs* or ds*GFP* was injected into 5-day-old *B. dorsalis* adults using a Nanoject II Auto-Nanoliter Injector (Drummond Scientific, Broomall, PA, USA). Tested flies (male to female ratio 1:1) were collected at 24 h after injection and total RNAs were isolated for gene silencing efficiency determination. Another batch of dsRNA-injected flies were collected and injected with living *E. coli* (Gram-negative) and *S. aureus* (Gram-positive) after 24 h. Each insect was injected with 200 nL *E. coli* solution with an OD_600_ value of 2.0 and *S. aureus* with an OD_600_ value of 1.0. Control flies were injected with 200 nL of PBS solution. In each group, a total of 60 flies were injected and their survival rates were recorded for at least 5 days.

We also evaluated the AMPs gene expression after gene silencing followed by bacterial infection. The alive bacteria were inactivated by incubating at 65 °C for 10 min. Flies were injected with inactivated *E. coli* and *S. aureus*, at 24 h after dsRNA injection. The control flies were injected with 200 nL of PBS. The flies were collected at 24 h after inactive pathogens injection for transcriptional analysis. The relative expression level of target genes and four downstream AMPs genes, *attacin-A* (GenBank accession number: KY038167), *cecropin-2* and *defensin* (GenBank accession number: KX510001 and KX510002) [35], and *diptericin* (GenBank accession number of KJ488999) [36], were selected for gene expression determination by RT-qPCR.

### 2.8. Statistical Analysis

All of the experiments were conducted with three biological replications on the same day. The differences of gene expression levels were analyzed using one-way analysis of variance (ANOVA), followed by Tukey’s honestly significant difference (HSD) multiple comparison test using SPSS ver. 19.0 software (IBM, Chicago, IL, USA). The differences between pathogen treatment and silencing efficiency were determined by the independent sample Student’s *t* test (*p* < 0.05).

## 3. Results

### 3.1. Sequence Analysis, Alignments, and Phylogenetic Trees

**Figure 1:**
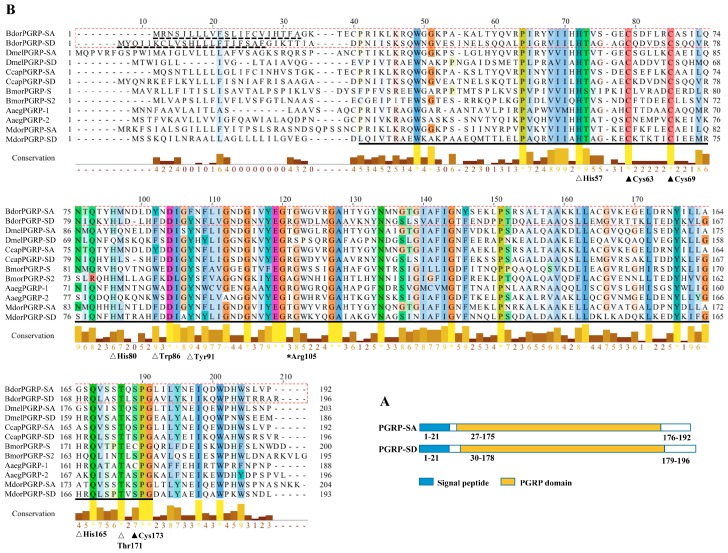
Structure of the signal peptide and conserved domain of two BdPGRPs (**A**), and multiple sequence alignment of PGRPs from insects (**B**). The blue bar and yellow bars in panel A indicate the signal peptide and conserved PGRP domain, respectively. The dashed line indicates the signal peptide amino acids, and the black line indicates the conserved PGRP domain region. The black triangles in panel B indicate the conserved cysteine residues, the clear triangles indicate the amino acid residues required for PGRP/amidase activity, and the asterisk indicates the amino acid responsible for the recognition of DAP-PGN. The heights of the yellow or brown bars below the aligned sequences represent the degree of similarity of the amino acids. Bdor, Dmel, Ccap, Bmor, Aaeg, and Mdom indicate the insects of *Bactrocera dorsalis*, *Drosophila melanogaster*, *Ceratitis capitata*, *Bombyx mori*, *Aedes aegypti*, and *Musca domestica*, respectively.

The cDNA sequences of *BdPGRP-SA* and *BdPGRP-SD* were cloned from adult female *B. dorsalis*. The full-length cDNA sequence of *BdPGRP-SA* and *BdPGRP-SD* contained an open reading frame of 579 bp and 591 bp, encoding 192 and 196 amino acid residues, respectively. Both genes were uploaded to NCBI GenBank with accession numbers of MK392505 and MK392506. Based on the conserved domain search result on NCBI, *BdPGRP-SA* and *BdPGRP-SD* were both expected to be members of the PGRP superfamily. According to SignalP 4.0, predicted signal peptides for PGRP-SA and PGRP-SD were 21 and 21 amino acids, respectively, but no transmembrane domain was predicted (Figure 1A). Alignment was carried out using the Clustal Omega program and visualized using Jalview software 2.9 (http://www.mybiosoftware.com/jalview-2-6-1-multiple-alignment-editor.html) (Figure 1B). Only BdPGRP-SD has an Arg105 amino acid residue, which was required for the recognition of DAP-PGN. The two conserved cysteines were determined in both sequences. Comparative analysis indicated that the amino acids of His57, Tyr91, Thr171, which are required for T7 lysozyme Zn^2+^ binding and amidase activity, were conserved in *BdPGRP-SD* (Figure 1B). The Trp86 was not in both sequences, indicating that one of the PGRPs was catalytic PGRPs.

**Figure 2:**
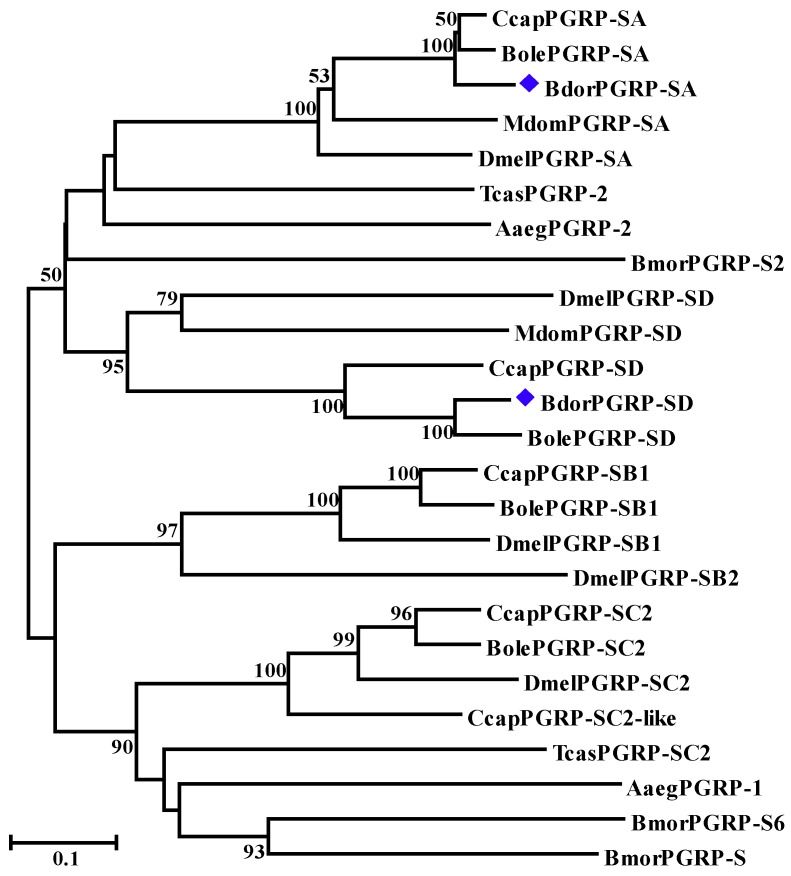
Phylogenetic analysis of the insect PGRPs. MEGA 5 was used to construct the phylogenetic tree using the neighbor-joining method with 1000 bootstrap replicates. All of the homologous PGRPs were retrieved from the NCBI. Insect species include *Drosophila melanogaster* (Dmel), *Aedes aegypti* (Aaeg), *Bactrocera oleae* (Bole), *Ceratitis capitata* (Ccap), *Musca domestica* (Mdom), and *Tribolium castaneum* (Tcas). Table S2 shows the amino acid sequences of the PGRPs used in the phylogenetic tree.

A phylogenetic tree was constructed using the neighbor-joining method to analyze the relationships among PGRP-SA and PGRP-SD proteins in *B. dorsalis* with other insect PGRPs. BdPGRP-SA and BdPGRP-SD clustered into two separate clades that are the same clades as their orthologues homologs in other species (Figure 2). BdPGRP-SA was closely related to the PGRP-SA in *C. capitata* and *Bactrocera oleae*. However, BdPGRP-SD was closely related to the PGRP-SD in *B. oleae*.

### 3.2. Spatiotemporal Expression Analysis

**Figure 3:**
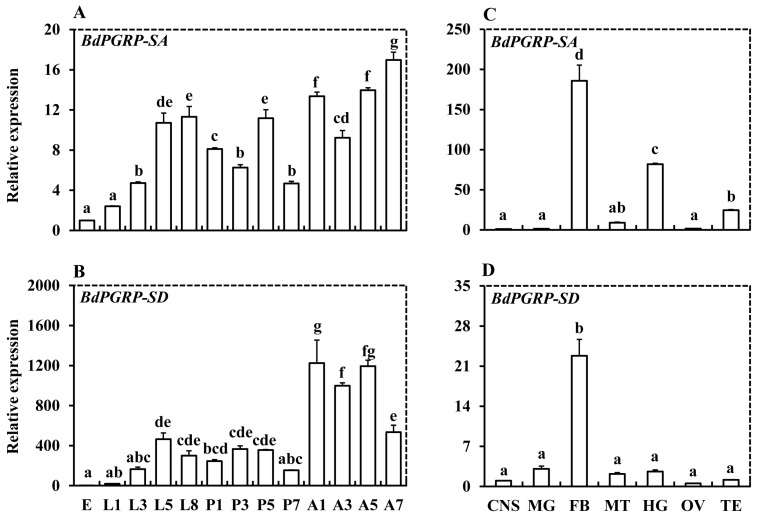
Gene expression profiles of *BdPGRP-SA* and *BdPGRp-SD* in the different developmental stages (**A** and **B**), and tissues of 5-day-old *Bactrocera dorsalis* adults (**C** and **D**). Egg (E); larvae on days 1, 3, 5, and 8 (L1, L3, L5, L8); pupae on days 1, 3, 5, and 7 (P1, P3, P5, P7); and adults on days 1, 3, 5, and 7 (A1, A3, A5, A7) of *B. dorsalis* were sampled for developmental stage expression analysis. Central nervous (CNS), midgut (MG), hindgut (HG), fat body (FB), Malpighian tubule (MT), ovary (OV), and testis (TE) in 5-day-old adults were collected for tissue expression determination. Different letters above the error bars indicate statistical differences (*p* < 0.05).

We determined the expression of *BdPGRP-SA* and -*SD* in the developmental stages and tissues of *B. dorsalis* using qRT-PCR. *BdPGRP-SA* was expressed in all of the developmental stages (Figure 3A). The expression of *BdPGRP-SA* increased during the larval development [37]. The expression of *BdPGRP-SD* was significantly higher in adults (Figure 3B). Among the different tissues of adult flies, *BdPGRP-SA* was highly expressed in fat body and hindgut (Figure 3C). *BdPGRP-SD* was highly expressed only in fat body, but had low expression in other tissues (Figure 3D).

### 3.3. Expression Induced by Microbial Challenges

**Figure 4:**
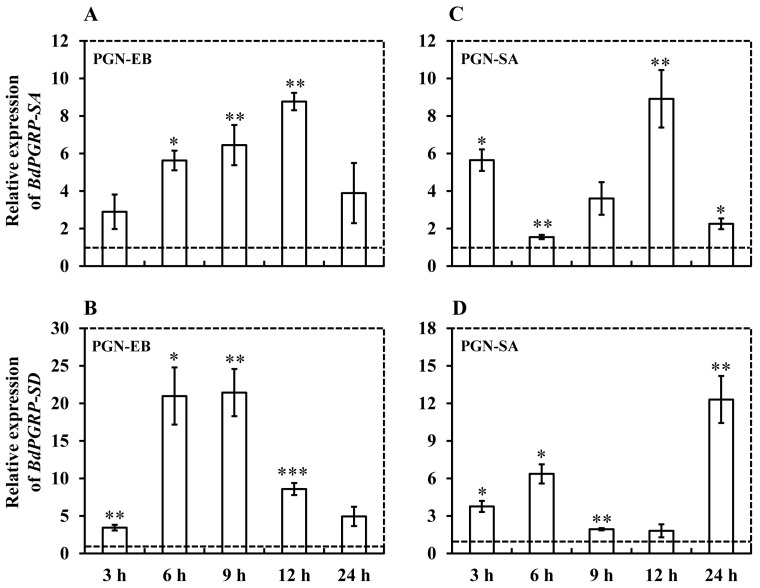
The expression of *BdPGRP-SA* and *BdPGRp-SD* in 5-day-old adults injected with peptidoglycan of PGN-EB and PGN-SA. PGN-EB and PGN-SA represents peptidoglycan from *Escherichia coli* 0111:B4 and *Staphylococcus aureus*, respectively. The control group was injected with an equal amount of PBS. Two flies (one male and one female) were collected and pooled at 3, 6, 9, 12, and 24 h post-pathogen injection. Significant differences were determined via the independent samples *t*-test (* indicates *p* < 0.05, ** indicates *p* < 0.01, and *** indicates *p* < 0.001).

To study the potential role of *BdPGRP-SA* and -*SD* in *B. dorsalis* immunity, 5-day-old adults were injected with the PGN-EB and PGN-SA. *BdPGRP-SA* and *-SD* were up-regulated to a peak at 12 h and 9 h respectively, and then decreased to a normal level at 24 h when challenged by PGN-EB (Figure 4A,B). When challenged by PGN-SA, the expression of *BdPGRP-SA* and *BdPGRP-SD* were up-regulated with a dynamic fluctuation. *BdPPGRP-SA* was up-regulated to be bi-modal with one peak at 3 h and a second at 12 h (Figure 4C). While *BdPGRP-SD* showed a similar profile but with a first peak at 6 h and a second at 24 h (Figure 4D).

### 3.4. RNAi Bioassay

**Figure 5:**
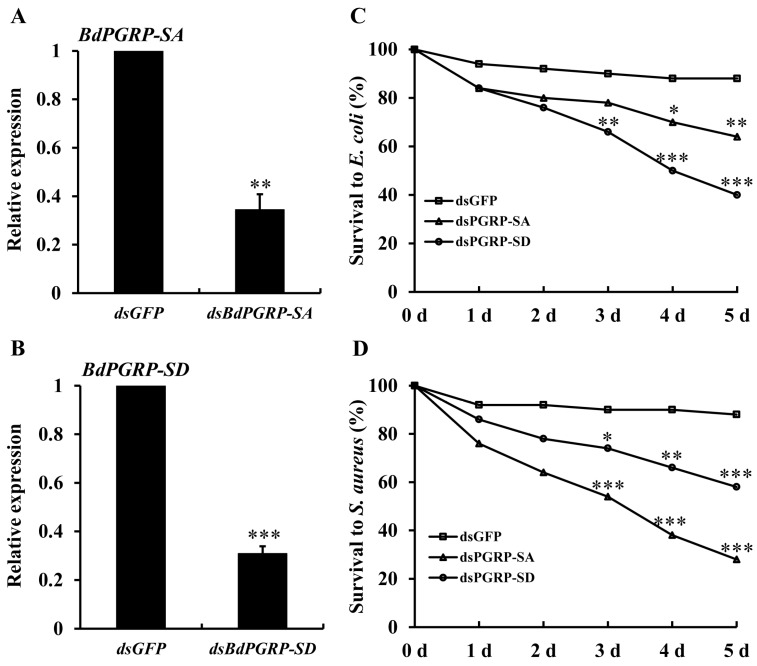
Relative transcriptional expression of *BdPGPR-SA* and *BdPGRP-SD* after gene-specific dsRNA injection (**A** and **B**), and the survival of flies after dsRNA injection followed by *E. coli* and *S. aureus* injection (**C** and **D**). The gene silencing efficiency was detected at 24 h after injection. Survival rates were calculated within 5 days after gene-specific dsRNA injection and *E. coli* and *S. aureus* 24 h later. Same amounts of dsGFP was injected as the negative control. Significant differences were determined by the independent samples *t*-test (* indicates *p* < 0.05, ** indicates *p* < 0.01, and *** indicates *p* < 0.001).

At 24 h after injection of gene-specific dsRNA, the transcriptional expression of *BdPGRP-SA* and *-SD* decreased by 65.5% and 69.1%, respectively (Figure 5A,B). When injected with *E. coli* and *S. aureus*, the dsBdPGRP-SA and -SD injected flies showed significantly lower survival rates within 5 days (Figure 5C,D). Survival at 5 days in the dsGFP groups was 88% when injected with *E. coli*. Survival of the dsBdPGRP-SA and -SD groups was significantly lower at 64% and 40%, respectively. In the *S. aureus* injection group, the survival rate of dsGFP was 88%, while the survival of ds*BdPGRP-SA* and *-SD* were 28% and 59%, respectively. *BdPGRP-SD* was more sensitive to *E. coli* and the *BdPGRP-SA* was more sensitive to *S. aureus*.

### 3.5. Expression of Antimicrobial Peptides (AMPs)

**Figure 6:**
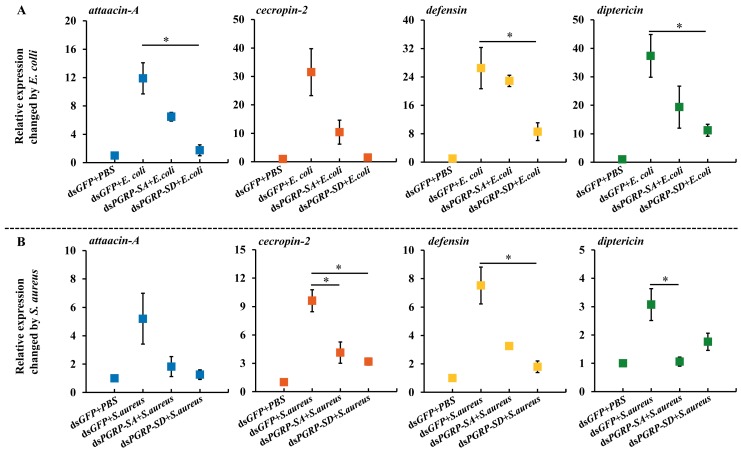
Relative expression level of antibacterial peptides *attacin-A*, *cecropin-2*, *defensin*, and *diptercin* after injection with dsRNA and injection with inactivated *Escherichia coli* (**A**) and *Staphylococcus aureus* (**B**). The data represent mean ± SE. Significant differences determined by the independent samples *t*-test (* indicates *p* < 0.05).

Flies injected for gene-specific suppression were collected for microbial injection at 24 h. The transcription level of four AMPs genes, including *attacin-A*, *cecropin-2*, *defensin*, and *diptericin*, were determined using RT-qPCR. The gene expressions of the four AMPs were significantly up-regulated by Gram-negative and Gram-positive bacteria (Figure 6). In the *E. coli* treatment, the expressions of the four AMPs genes—*attacin-A*, *cecropin-2*, *defensin*, and *diptericin*—were induced to a high level when the *BdPGRP-SA* target gene was independently suppressed (Figure 6A). There was no difference between the *BdPGRP-SA* dsRNA injection group and the control. In the *BdPGRP-SD* dsRNA injection, three AMPs, including *attacin-A*, *defensin*, and *diptericin*, were not induced, and showed significantly lower expression than the negative control. This indicated that it was BdPGRP-SD, not BdPGRP-SA, that recognized the Gram-negative pathogen (i.e., *E. coli*) infection and responded by activating the expression of three AMPs. A similar trend of *cecropin-2* expression also occurred in response to the *E. coli* infection. When flies were challenged by *S. aureus*, two downstream AMPs (*cecropin-2* and *diptericin*) were expressed at a significantly lower level after *BdPGRP-SA* silencing (Figure 6B). This indicates that *cecropin-2* and *diptericin* could be induced via the recognition of *BdPGRP-SA* against Gram-positive bacteria. When injected with *S. aureus*, *cecropin-2* and *defensin* were expressed at a lower level in *BdPGRP-SD* suppressed flies, indicating that *BdPGRP-SD* was also involved in response to Gram-positive bacteria (Figure 6B).

## 4. Discussion

Insects rely on innate immunity for defense against microbial pathogens. Defenses include both the inducible AMPs and the activated prophenoloxidase cascades in the hemolymph [38]. In insects, peptidoglycan recognition proteins (PGRPs) play key roles in recognizing bacterial infection [7]. In this study, we cloned the transcripts of *BdPGRP-SA* and *BdPGRP-SD* found in *B. dorsalis*. The Arg amino acid residue was only found in BdPGRP-SD sequence and this is required for the recognition of DAP-PGN. His, Tyr, and Thr amino acids, required for T7 lysozyme Zn^2+^ binding and amidase activity, were partially conserved in two *BdPGRP-Ss*. For example, Tyr was replaced by Phe residue in *BdPGRP-SA*. The catalytic domain was associated with Gram-negative binding protein 1 (GNBP1) in activating the Toll receptor pathway [39]. Both genes may be involved in different pathways. In *Drosophila*, the PGRP-SA and -SD can discriminate different bacteria containing either Lys-PGN and DAP-PGN to elicit down-stream antimicrobial responses via activating the Toll and Imd pathways respectively [7]. In *Antheraea pernyi*, the conserved Tyr was also replaced by another amino acid, indicating a pattern recognition receptor in the immune system [40]. The amino acid Trp was also not conserved in *B. dorsalis* in the alignment, but it is conserved in mosquitoes, e.g., *Aedes aegypti* [41]. These findings indicate non-catalytic PGRPs produced by two genes in *B. dorsalis*. Similarly, in *Drosophila*, the homolog PGRP-SA and -SD are non-catalytic PGRPs [42]. Phylogenetic tree comparison of the BdPGRP-SA and *-SD* with the homologs from other insects showed that the both proteins PGRPs are closely located with that from *B. oleae* and *M. domestica*.

Insect PGRP-Ss are present in the hemolymph and cuticle. They are constitutively synthesized or induced mainly in the fat body cells and sometimes in epidermal cells in the gut, and to a lesser extent, in hemocytes [43,44]. In *Drosophila*, *PGRP-SD* is moderately expressed in the fat body and midgut of adult flies and highly expressed in the fat body of larvae [25]. In this study, Bd*PGRP-SA* was highly expressed in both larvae and adults, and Bd*PGRP-SD* was highly expressed in adults. The fat body is an important immune organ that responds to pathogen infection, so it is reasonable that both genes were highly expressed in this tissue. In *Drosophila*, PGRP-SA and GNBP1 are secreted into the hemolymph and combine into a complex that activates a proteolytic cascade that culminates in the cleavage of Spätzle and the Toll signaling pathway [11,19].

Insect PGRPs are involved in many important signal pathways in the innate immune system. In *Drosophila*, PGRP-SA and -SD recognize bacterial PGN and activate the Toll receptor [19,20]. In *B. dorsalis*, the expression of *BdPGRP-SA* was significantly increased after induction by PGN-EB and PGN-SA. This indicated that *BdPGRP-SA* can be induced by *E. coli* and *S. aureus* (Figure 4). The inducible expression of PGRPs has been observed in *Armigeres subalbatus* [45], *A. aegypti* [41], and *Microplitis mediator* [46]. The induction of BdPGRP-SA and -SD by PGN-EB was confirmed earlier [47]. PGRP-SD was previously considered to be a pattern recognition receptor (PRR) for Gram-positive bacteria, but subsequent biochemical and structural analysis revealed that PGRP-SD holds a peptidoglycan-binding groove characteristic of DAP-type recognition PGRPs. An in vitro binding study demonstrated that PGRP-SD binds DAP-PGN but not Lys-PGN [24]. PGRP-SA is required for triggering the Toll pathway whereas PGRP-SD is not essential but enhances signaling. Toll activation initiates a signal transduction cascade that results in the expression of AMPs [39].

In *Drosophila*, all of the flies died within 3 days after Gram-positive bacteria, *S. aureus*, *Enterococcus faecalis*, and *Micrococcus luteus* injection when PGRP-SA was mutated [11,20]. Similarly, PGRP-SD mutants were sensitive to *S. aureus* and *S. pyogenes* [20]. The survival phenotype could be rescued by injection of recombinant PGRP-SD before being challenged with *S. aureus* [23]. When combined with the PGRP-SA mutant, this mutation further impaired expression of the Toll pathway target genes [20]. In the present study, after infection with *E. coli* and *S. aureus*, the mortality rate of *B. dorsalis* adults injected with dsBdPGRP-SA increased by 24% and 60%, respectively. This demonstrated that flies injected with dsBdPGRP-SA were more sensitive to *S. aureus*. The mortality of flies injected with ds*BdPGRP-SD* increased by 48% and 30% after *E. coli* and *S. aureus* infection, indicating a relatively higher sensibility to *E. coli* (Figure 5). The motilities of the two bacterial infections indicated that BdPGRP-SA was mainly involved in Gram-positive bacteria, possibly because of the replacement of the conserved Arg105 by a Thr. While BdPGRP-SD was involved in both Gram-negative and positive bacteria. A similar result was found in *Drosophila* where PGRP-SD mutants had increased susceptibility to some DAP-PGN bacteria species [12]. In *Drosophila*, *PGRP-SD* mutants had a reduced systemic Imd pathway activation and increased susceptibility to Gram-negative bacteria [25]. This revealed a new regulating Imd signaling pathway instead of the Toll pathways. The new functional mechanism was thereafter covered to enhance peptidoglycan-mediated activation of the Imd pathway by promoting peptidoglycan re-localization to the cell-surface receptor PGRP-LC [25].

Numerous AMPs have been identified, but their production pathways are usually unclear. When challenged by *Erwinia carotovora* or *E. coli*, the expression levels of AMPs transcripts remained at a high level in *Drosophila* [11,48]. Similarly, the transcription level of AMP genes, including *attacin*, *cecropin,* and *lysozyme*, were greatly up-regulated following the infection of *E. coli* and *S. aureus*, but they decreased when endogenous *PGRP-SA* was knocked down in *A. pernyi* [41]. Our data showed that *E. coli* induced a high expression of AMPs, but silencing of *BdPGRP-SA* did not result in low expression of the AMPs. When challenged by *S. aureus*, *cecropin-2* and *diptericin* were inhibited in *dsBdPGRP-SA* injected flies. This indicated a role of BdPGRP-SA in response to Gram-positive bacterial infection by activating downstream AMPs expression, e.g., *cecropin-2* and *diptericin*. Similarly, when *PGRP-SA* was suppressed in *A. pernyi,* the expression of *cecropin-B* was not induced, i.e., exactly one fifth, after challenge by *E. coli* [41]. Attacin and diptericin (an anti-Gram-negative bacterial peptide) are known to be involved in the Imd pathway, and cecropin and defensin are common AMPs in insects and involved in *B. dorsalis* immunity [35]. The present study on *B. dorsalis* demonstrated the important immune role of *BdPGRP-SA* in recognizing DAP-PGN and activating the production of AMPs against pathogen infection. High expression of all four AMPs was induced by Gram-negative *E. coli* and three of them were inhibited by ds*BdPGRP-SD*. However, no changes in AMPs expression occurred when *BdPGRP-SA* was silenced. BdPGRP-SD also plays an important role in response to Gram-negative bacteria infection. In a previous study, *diptericin* was induced via Gram-negative bacteria in *B. dorsalis* [36], possibly because of recognition by BdPGRP-SD. The induction by PGN-SA and inhibition of *cecropin* and *defensin* by exogenous dsBdPGRP-SD indicates its role in recognition of Gram-positive bacteria. Similarly, the PGRP-SD mutant is more sensitive to *S. aureus* in *Drosophila* [20].

## 5. Conclusions

We cloned two PGRP genes, *BdPGRP-SA* and *BdPGRP-SD* in *B. dorsalis*, and determined their roles in response to Gram-negative and Gram-positive pathogen infection. Both genes were highly expressed in adult fat body. Their inducible expressions by PGN indicated potential functions and these were validated using RNAi analysis and AMPs gene expression. In the innate immune system of *B. dorsalis*, BdPGRP-SA plays an essential role in response against Gram-positive pathogens, and BdPGRP-SD is important in response to Gram-negative pathogens, as well as Gram-positive pathogens. They act with important roles, in different ways, by regulating downstream AMPs gene expression.

## Supplementary Materials

The following are available online at https://www.mdpi.com/2075-4450/10/3/79/s1, Table S1: Primer sequence used for cloning and quantitative real-time PCR, Table S2: Amino sequences of PGRPs used in the phylogenetic tree.
